# Deficits in Motor Performance, Neurotransmitters and Synaptic Plasticity in Elderly and Experimental Parkinsonian Mice Lacking GPR37

**DOI:** 10.3389/fnagi.2020.00084

**Published:** 2020-03-27

**Authors:** Xiaoqun Zhang, Ioannis Mantas, Elva Fridjonsdottir, Per E. Andrén, Karima Chergui, Per Svenningsson

**Affiliations:** ^1^Laboratory of Translational Neuropharmacology, Department of Clinical Neuroscience, Karolinska Institutet, Stockholm, Sweden; ^2^Medical Mass Spectrometry Imaging, Department of Pharmaceutical Biosciences, Uppsala University, Uppsala, Sweden; ^3^National Resource for Mass Spectrometry Imaging, Science for Life Laboratory, Uppsala University, Uppsala, Sweden; ^4^Laboratory of Molecular Neurophysiology, Department of Physiology and Pharmacology, Karolinska Institutet, Stockholm, Sweden

**Keywords:** Parkinson’s disease, 6-hydroxydopamine, striatum, synaptic plasticity, GPR37, PAEL-R

## Abstract

Parkinson’s disease (PD) etiology is attributed to aging and the progressive neurodegeneration of dopamine (DA) neurons of substantia nigra pars compacta (SNc). GPR37 is an orphan G-protein Coupled Receptor (GPCR) that is linked to the juvenile form of PD. In addition, misfolded GPR37 has been found in Lewy bodies. However, properly folded GPR37 found at the cell membrane appears to exert neuroprotection. In the present study we investigated the role of GPR37 in motor deficits due to aging or toxin-induced experimental parkinsonism. Elderly GPR37 knock out (KO) mice displayed hypolocomotion and worse fine movement performance compared to their WT counterparts. Striatal slice electrophysiology reveiled that GPR37 KO mice show profound decrease in long term potentiation (LTP) formation which is accompanied by an alteration in glutamate receptor subunit content. GPR37 KO animals exposed to intrastriatal 6-hydroxydopamine (6-OHDA) show poorer score in the behavioral cylinder test and more loss of the DA transporter (DAT) in striatum. The GPR37 KO striata exhibit a significant increase in GABA which is aggravated after DA depletion. Our data indicate that GPR37 KO mice have DA neuron deficit, enhanced striatal GABA levels and deficient corticostriatal LTP. They also respond stronger to 6-OHDA-induced neurotoxicity. Taken together, the data indicate that properly functional GPR37 may counteract aging processes and parkinsonism.

## Introduction

The most common risk factor to develop Parkinson’s disease (PD) is aging (Poewe et al., 2017). A neuropathological hallmark of Parkinson’s disease (PD) is the slow and progressive degeneration of the dopamine (DA) producing neurons located in substantia nigra pars compacta (SNc) (Ehringer and Hornykiewicz, 1998; Poewe et al., 2017). The motor symptoms of PD patients is attributed to the reduced dopamine levels and the inability of activation of striatal D1 and D2 DA receptors (Gerfen, 2000). The expression of these receptors is segregated in the stritatonigral and striatopallidal subtypes of medium spiny neurons (MSNs), which compose the most abundant cell type in striatum (Gerfen et al., 1990; Surmeier et al., 1996). Several studies have shown that striata in the PD state display specific alterations indicative of the DA-depletion (McGregor and Nelson, 2019). For example, DA neuron deafferentation leads to profound impairments in corticostriatal plasticity and increased reciprocal GABA release (Calabresi et al., 2007; Lalchandani et al., 2013; Wei et al., 2017). The mechanisms underlying the progressive dopaminergic neurodegeneration in PD remains largely unknown, but several studies emphasize lysosomal dysfunction, abnormal accumulation of misfolded proteins and oxidative stress (Surmeier et al., 2017). The most well-known protein to aggregate in PD is alpha-synuclein, which is the major constituent of Lewy body inclusions (Spillantini et al., 1997; Surmeier et al., 2017). There are mutations and multiplications of the alpha-synuclein gene in some familial PD along with several other genes which cause autosomal recessive or dominant PD (Singleton et al., 2017). The *parkin* gene is mutated in autosomal recessive juvenile parkinsonism (ARJP) (Kitada et al., 1998; Giasson and Lee, 2001). Parkin is an E3 ubiquitin ligase that regulates mitochondrial autophagy (Kitada et al., 1998; Giasson and Lee, 2001). GPR37 is an orphan G-protein coupled receptor (GPCR) which has been described as a target of Parkin and is reported to be found within Lewy bodies in PD brains (Murakami et al., 2004). GPR37 is also named parkin associated endothelin B-like receptor (PAEL-R) due to its relationship with parkin and its homology with the endothelin B receptor (ETBR) (Leinartaité and Svenningsson, 2017). Despite its resemblance with the ETBR, the endogenous ligand of GPR37 remains to be identified (Leinartaité and Svenningsson, 2017). GPR37 trafficking from endoplasmic reticulum to the plasma membrane plays a crucial role for neuronal viability (Lundius et al., 2014). Specifically, the surface expression of the receptor displays neuroprotective properties while the intracellular retention of the protein leads to misfolding, aggregation, and subsequent degeneration (Leinartaité and Svenningsson, 2017). Moreover, forced overexpression of the receptor leads to profound neurodegeneration in animal models, which shows selectivity for DA neurons (Dusonchet et al., 2009). GPR37 shows features of a double-edged sword that simultaneously can support or hamper neuronal viability depending upon its folding and cellular localization.

In the present study, we used GPR37 knock out (KO) mice and studied motor performance upon aging. Electrophysiological studies in striatal slices examined long-term potentiation (LTP), known to be modulated by dopamine and glutamate interactions. Finally, behavioral and neurotransmitter alterations upon intrastriatal 6-hydroxydopamine (6-OHDA) administration were examined in GPR37 KO mice.

## Materials and Methods

### Animals

The experiments were approved by the local ethical committee at Karolinska Institute (N105/16) and conducted in accordance with the European Communities Council Directive of November 24, 1986 (86/609/EEC). Wildtype (WT) and GPR37 KO mice on a C57Bl6 background were used (Meyer et al., 2013; kindly provided by Dr. Randy Hall). They were housed in temperature- and humidity-controlled rooms (20°C, 53% humidity) with a 12 h dark/light cycle. They had access to standard lab pellets and water *ad libitum*.

### Behavioral Tests

Adult (4–5 months) and old (11 months) mice were tested for exploratory locomotion behavior, posture control and coordination. Mice were randomized according to genotype and tested at each age with a 6-days interval between the behavioral tests. All behavioral tests were performed during the light cycle by experimenters blind to the genotype. Mice were allowed to habituate to the experimental room for at least 1 h before each test. Behavioral equipment was cleaned with 70% ethanol after each test session to avoid olfactory cues.

#### Open Field

The test was performed for 30 min in a 46 cm × 46 cm arena illuminated with a 30 lux light. Video tracking was performed using a video camera mounted in the ceiling coupled to the EthoVision XT11.5 (Noduls) software.

#### Pole Test

Mice were placed head-up on top of a vertical pole (diameter: 8 mm, height: 55 cm) and trained 1 day to turn and descend the pole back into the cage. On the day of the test, animals performed three trials. The time to orient downward (t-turn) and the total time to turn and descend the pole were measured, with a maximum duration of 120 s.

#### Beam Traversal Test

The beam was constructed as described (Fleming et al., 2004). Mice were trained for two consecutive days to traverse the beam. On the test day a grid (1 cm^2^) of corresponding width was placed 1cm above the beam, mice were videotaped while traversing it for a total of five trials, and steps were counted.

#### Cylinder Test

Mice were placed in a transparent glass cylinder (13 cm diameter, 24 cm height) to examine forelimb use. We counted the number of times the mice touched the wall of the cylinder with their left forepaw (contralateral to the lesion) and right forepaw (ipsilateral to the lesion) during 5 min to evaluate forelimb use asymmetry.

### Intrastriatal 6-OHDA

Adult GPR37 KO and WT mice (4–6 months of age) were pretreated with 25 mg/kg desipramine (i.p.; Sigma-Aldrich), a noradrenergic reuptake inhibitor, and 5 mg/kg pargyline (i.p.; Sigma-Aldrich), a monoamine oxidase B inhibitor, to reduce any possible breakdown of the toxin by the enzyme (Duty and Jenner, 2011). After 30 min, they were anesthetized (80 mg/kg ketamine and 5 mg/kg xylazine, i.p.), mounted in a stereotaxic frame and unilaterally injected with 10 μg 6-OHDA (Sigma-Aldrich Co. LLC, St. Louis, MO, United States) (10 μg/μl in saline containing 0.02% ascorbic acid) into the striatum of the right hemisphere to produce partial lesions. The coordinates for injections were: AP, + 0.5 mm; ML, -2 mm; and DV, -2.8 mm relative to bregma and the dural surface (Gould et al., 2012). Two weeks after 6-OHDA lesioning, mice were administered 1 mg/kg apomorphine (i.p.; Sigma-Aldrich) and rotations were counted for 30 min. Mice with intrastriatal 6-OHDA lesions were sacrificed 4 weeks after surgery and the brains were dissected out and cut in the cryostat. The slides that were closer to the bregma (+0.5 mm), where 6-OHDA was injected, were used for autoradiography and matrix-assisted laser desorption ionization mass spectrometry imaging (MALDI-MSI).

### In situ Hybridization (ISH)

A ^35^S-labeled anti-sense cRNA probe against *GPR37* was prepared by *in vitro* transcription from a cDNA clone corresponding to fragments of *GPR37.* The transcription was performed from 100 ng of linearized plasmid using ^35^S-UTP (1000 Ci/mmol) and T3 RNA polymerase. *In situ* hybridization was performed as previously described (Zhang et al., 2007). Briefly, 12 μm thick cryostat sections were postfixed in 4% PFA for 5 min at room temperature, rinsed twice in 4 × sodium chloride–sodium citrate buffer (SSC) and placed into 0.25% acetic anhydride in 0.1 M triethanolamine/4 × SSC (pH 8) for 10 min at room temperature. After dehydration in graded alcohols, the sections were hybridized overnight at 55°C with ^35^S-labeled *GPR37* probe in 50 μl of hybridization solution (20 mM Tris–HCl/1 mM EDTA/300 mM NaCl/50% formamide/10% dextran sulfate/1 × Denhardt’s/250 μg/ml yeast tRNA/100 μg/ml salmon sperm DNA/0.1% SDS/0.1% sodium thiosulphate). The slides were washed in 4 × SSC (5 min, four times), RNAse A (20 μg/ml) (20 min, at 37°C), 2 × SSC (5 min, twice), 1 × SSC (5 min), 0.5 × SSC (5 min, twice) at room temperature, and rinsed in 0.1 × SSC at 65°C (30 min, twice) (all washes contained 1 mM DTT), before being dehydrated in graded alcohols. The slides were then exposed on X-ray films for 4 to 28 days. Autoradiograms were digitized using a Dia-Scanner (Epson Perfection 4870 PHOTO). Optical density values were measured using Image J (1.52 h, Wayne Rasband National Institutes of Health, United States).

### Autoradiographic Detection of DAT

Frozen brains were cryostat cut in 12 μm sections and mounted on SuperFrost microscope slides (Gerhard Menzel GmbH, Braunschweig, Germany) for DAT binding by autoradiography. Sections were preincubated in 50 mM Tris–HCl/120 mM NaCl (pH 7.5) for 20 min, and then incubated in binding buffer with 50 pM [^125^I] RTI-55 (Perkin-Elmer Life Sciences, Boston, United States) with 1 μM fluoxetine (Tocris Bioscience, Bristol, United Kingdom) for 60 min. 100 μM nomifensine (Sigma-Aldrich) was added to the assay to determine non-specific binding. The slides were washed in ice-cold binding buffer (2 × 10 s) and in deionized water, dried, and exposed to Kodak Biomax MR film (Sigma-Aldrich). Autoradiograms from ligand binding autoradiography were digitized using a Dia-Scanner (Epson Perfection 4870 PHOTO). Optical density values were measured using Image J (1.52 h, Wayne Rasband National Institutes of Health, United States).

### Striatal Slice Electrophysiology

Adult GPR37 KO and WT mice (4–6 months of age) underwent cervical dislocation followed by decapitation. Their brains were rapidly removed, and coronal brain slices (400 μm thick) containing the striatum and the overlying cortex were prepared with a microslicer (VT 1000S; Leica Microsystem, Heppenheim, Germany). Slices were incubated, for at least 1 h, at 32°C in oxygenated (95% O_2_ + 5% CO_2_) artificial cerebrospinal fluid (aCSF) containing (in mmol/L): 126 NaCl, 2.5 KCl, 1.2 NaH_2_PO_4_, 1.3 MgCl_2_, 2.4 CaCl_2_, 10 glucose, and 26 NaHCO_3_, pH 7.4. Slices were transferred to a recording chamber and were continuously perfused with oxygenated aCSF at 28°C. Data were acquired and analyzed with the pClamp 9 or pClamp 10 software (Axon Instruments, Foster City, CA, United States). Extracellular field potentials were recorded using a glass micropipette filled with aCSF positioned on the slice surface in the dorsolateral part of the striatum. These synaptic responses were evoked by stimulation pulses applied every 15 s to the brain slice through a concentric bipolar stimulating electrode (FHC, Bowdoinham, ME, United States) placed near the recording electrode on the surface of the slice. Single stimuli (0.1 ms duration) were applied at an intensity yielding 50–60% maximal response as assessed by a stimulus/response curve established, for each slice, at the beginning of the recording session, by measuring the amplitude of the field excitatory postsynaptic potentials/population spikes (fEPSP/PSs) evoked by increasing stimulation intensities. High-frequency stimulation (HFS) was used to induce LTP of the fEPSP/PS and consisted of 100−Hz trains of 1-s duration repeated 4 times with a 10-s inter-train interval. Signals were amplified 500 or 1000 times via an Axopatch 200B or a GeneClamp 500B amplifier (Axon Instruments), acquired at 10 kHz and filtered at 2 kHz.

### Western Blotting

The striata tissue from adult WT and GPR37 KO mice were sonicated in 1% SDS and boiled for 10 min. Protein concentration was determined in each sample with a bicinchoninic acid protein assay (BCA-kit, Pierce, Rockford, IL, United States). Equal amounts of protein (30 μg) were re-suspended in sample buffer and separated by SDS-PAGE using a 10% running gel and transferred to an Immobilon-P (polyvinylidene difluoride) transfer membrane (Sigma-Aldrich). The membranes were incubated for 1 h at room temperature with 5% (w/v) dry milk in TBS–Tween20. Immunoblotting was carried out with antibodies against Ser^845^-GluA1, Ser^896^-GluN1, Ser^897^-GluN1, Ser^1303^-GluN2B, total GluA1, total GluN1 and total GluN2B (Calbiochem, Merck, Darmstadt, Germany), in 5% dry milk dissolved in TBS-Tween 20 for 2 h at room temperature. The membranes were washed three times with TBS–Tween20 and incubated with secondary HRP-linked anti-rabbit IgG (H + L) (Thermo Fisher Scientific, Göteborg, Sweden; 1:6000 dilution) for 1 h at room temperature. At the end of the incubation, membranes were washed six times with TBS–Tween 20 and immunoreactive bands were detected by enhanced chemiluminescence (PerkinElmer, Waltham, MA, United States). Autoradiograms were digitized using a Dia-Scanner (Epson Perfection 4870 PHOTO). Optical density values were measured using Image J (1.52 h, Wayne Rasband National Institutes of Health, United States). The signal of every band was normalized to β-actin signal intensity. The bands for the phosphorylated protein forms of NMDA and AMPA were normalized to the total NMDA and AMPA protein.

### MALDI-MSI of Neurotransmitters and Metabolites

Brain tissue sections collected for MALDI-MSI were thaw-mounted on indium tin oxide coated glass slides (Bruker Daltonics, Bremen, Germany). For MALDI-MSI of neurotransmitters and metabolites on-tissue derivatization was performed according to a previously published protocol (Shariatgorji et al., 2019). In brief, a solution containing 1.8 mg/mL of FMP-10 in 70% ACN was applied in 30 passes at a flow rate of 80 μL/min using a TM-sprayer (HTX-technologies, Carrbooro, NC, United States) with the following parameters: 80°C spray temperature, 1100 mm/min spray velocity, 2.0 mm track spacing, and 6 psi nitrogen pressure.

MALDI-MSI was performed in positive ion mode on a MALDI TOF/TOF instrument (ultrafleXtreme, Bruker Daltonics) equipped with a smartbeam-II 2 kHz Nd:YAG laser. Data were collected in the mass range *m/z* 300–1000 at a 100 μm lateral resolution. The laser power was optimized at the start of each run and 100 laser shots were fired per position. Samples were analyzed in a random order to avoid any bias due to changes in the instrument sensitivity or matrix degradation. MSI data were visualized using FlexImaging version 5.0 (Bruker Daltonics).

For relative quantitation, the MSI data were converted to msIQuant format via imzML. Regions were annotated manually in msIQuant version 2.0 (developed in-house, Källback et al., 2016). Total ion current (TIC) normalized average intensities of neurotransmitters and metabolites were exported from each region for statistical analysis.

### Statistical Analysis

Numerical values are expressed as means ± SEM. Behavioral data are expressed as absolute values whereas histological, electrophysiological and biochemical data are expressed as percent of the baseline.

Statistical significance of the results was assessed by using Student’s *t*-test or Mann-Whitney test for paired observations and one way or repeated measurements (RM) two way ANOVA for multiple comparison test followed by Fisher’s LSD *post hoc* test. *P* values less than 0.05 were considered significant. For all the statistical tests we used Graph Pad Prism 8.0.

## Results

### Validation of GPR37 KO by *in situ* Hybridization

**FIGURE 1:**
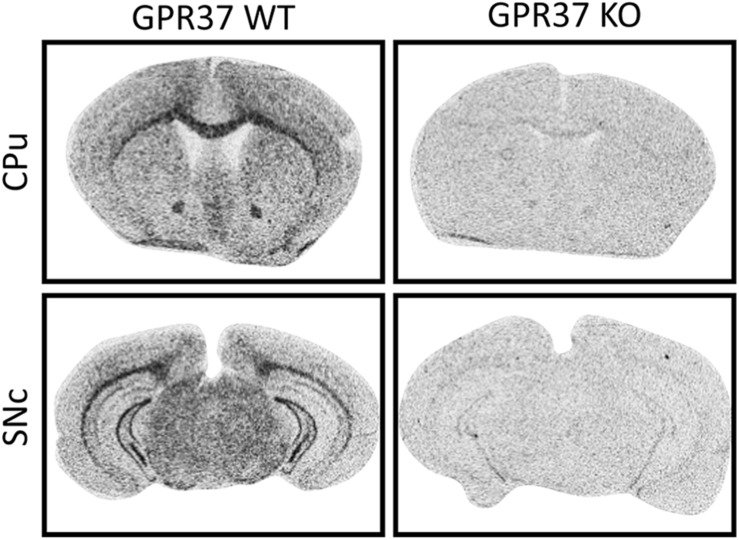
Validation of GPR37 KO by *in situ* hybridization mRNA expression of GPR37 in WT and GPR37KO mice at the levels of caudoputamen (CPu) and substantia nigra pars compacta (SNc).

The mouse line that we used is B6.129P2-*GPR37*^tm1Dgen^/J backcrossed onto a C57Bl6 background (Meyer et al., 2013). We validated the deletion of *GPR37* in the mouse line by radioactive ISH (Figure 1). ISH demonstrated widespread expression of GPR37 mRNA with enrichment in white matter tracts like corpus callosum which was absent in GPR37 KO mice (Figure 1; Yang et al., 2016; Morató et al., 2017; Bang et al., 2018; Jolly et al., 2018).

### GPR37 KO Mice Show Motor Deficits Upon Aging

**FIGURE 2:**
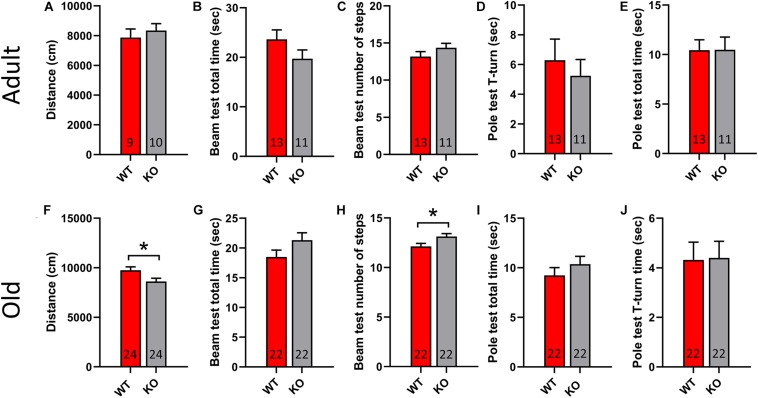
Old GPR37 KO display motor disturbances. **(A–E)** Bar graphs showing the motor performance of adult WT and GPR37 KO mice in open field **(A)**, beam test **(B,C)**, and pole test **(D,E)**. **(F–J)** Bar graphs showing the motor performance of old WT and GPR37 KO mice in open field **(F)**, beam test **(G,H)**, and pole test **(I,J)**. The *n* for each group is denoted in the corresponding bar. Data are expressed as mean ± SEM. *p < 0.05, Student’s *t*-test.

To study the motor performance of adult and old GPR37 KO mice we used a set of behavioral tests that are commonly used to assess PD animal models. Specifically, we used open field test, pole test and beam transversal test. Adult GPR37 KO animals do not show any significant difference to any test compared with the age-matched WT mice (Figures 2A–E). However, the elderly GPR37 KO mice traveled significantly less in open field than the WT mice (*p* = 0.02, Figure 2F). Furthermore, these GPR37 KO mice displayed significantly higher number of steps in the beam test (*p* = 0.02, Figure 2H). Neither adult nor old mice showed any significant difference in the pole test regarding the genotype factor (Figure 2). In summary, absence of GPR37 progressively leads to impaired motor function.

### Intrastriatal 6-OHDA Lesioned GPR37 KO Mice Show Fine Movement Impairments in Cylinder Test

**FIGURE 3:**
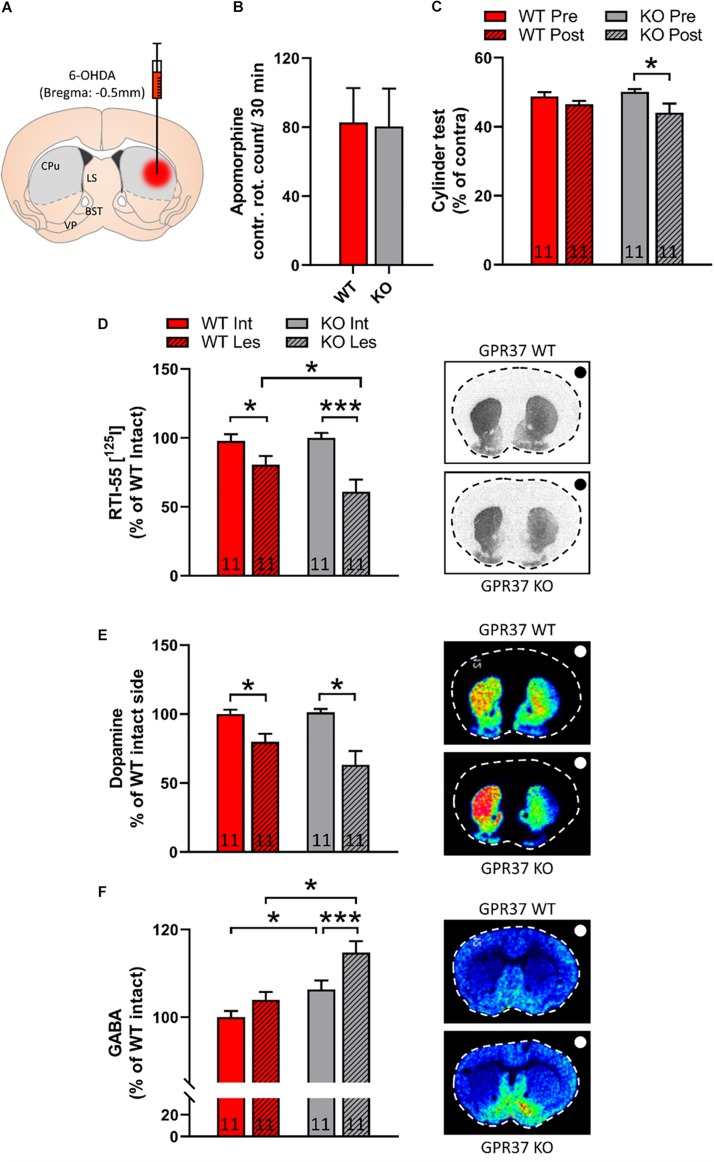
GPR37 KO show altered cylinder test score and striatal GABA after intrastriatal 6-OHDA lesion. **(A)** Schematic representation of intrastriatal 6-OHDA administration at bregma: + 0.5 mm. With gray it is depicted the region that was used for quantification. CPu, caudoputamen; LS, lateral septum; BST, bed nucleus of stria terminalis; VP, ventral pallidum. **(B)** Bar graph showing the contralateral rotation count after acute administration of apomorphine. **(C)** Bar graph showing the cylinder test score in WT and GPR37 KO mice, prior and after the 6-OHDA lesion. **(D)** Quantification of autoradiographic of specific striatal dopamine transporter binding with [^125^I] RTI-55. **(E,F)** Bar graphs showing the quantification of MALDI signal from dopamine (DA) **(E)** and GABA **(F)** in WT and GPR37 KO after the intrastriatal 6-OHDA lesion. The black/white dot on the right side of each picture indicates the side of the lesion. The *n* for each group is denoted in the corresponding bar. Data are expressed as mean ± SEM. *p < 0.05, **p < 0.01, and ***p < 0.001, Fisher’s LSD *post hoc* test.

To investigate the role of GPR37 in dopaminergic function we used the intrastriatal 6-OHDA PD animal model (Figure 3A). We did not observe any significant difference between the two genotypes in contralateral turns after acute apomorphine administration (Figure 3B). We performed cylinder test both in WT and GPR37 KO mice before and after 6-OHDA lesioning. RM two way ANOVA showed that there is significant effect of the pre-post lesion factor (Genotype: *p* = 0.73; Pre-post lesion: *p* = 0.01; Genotype × Pre-post lesion: *p* = 0.21, Figure 3C). *Post hoc* analysis revealed significant difference between pre and post lesion cylinder test score only in the GPR37 KO group (*p* = 0.02, Figure 3C).

### GPR37 KO Mice Show Enhanced Reduction of the Dopamine Transporter (DAT) Upon Intrastriatal 6-OHDA Lesioning

To address the possibility that the GPR37 KO mice show altered vulnerability toward 6-OHDA, we quantified DAT levels by using autoradiographic methods. The non-specific DAT binding is less than 3% of the specific. Hence, RM two way ANOVA performed upon DAT autoradiography data, showed that there is significant effect regarding the intact-lesion factor (Genotype: *p* = 0.22; Intact-lesion: *p* < 0.0001; Genotype × Intact-lesion: *p* = 0.06, Figure 3D). *Post hoc* analysis revealed significant 6-OHDA on both genotypes and significant difference between WT and GPR37 KO (WT intact vs. WT lesion: *p* = 0.04; KO intact vs. KO lesion: *p* < 0.0001; WT lesion vs. KO lesion: *p* = 0.03, Figure 3D). As DAT autoradiography is mostly depicting the dopaminergic axonal projection loss and not the levels of the dopamine release, we used MALDI-MSI to assess the striatal neurotransmitter content (Figure 3E). We observed that DA follow the same trend as DAT autoradiography signal, but the difference did not reach significance.

### GPR37 KO Mice Show Enhanced Levels of Striatal GABA Upon Intrastriatal 6-OHDA Lesioning

MSNs are GABAergic neurons and we used MALDI-MSI to measure striatal GABA content. RM two-way ANOVA unveiled significant effect of both genotype and intact-lesion factor (Genotype: *p* = 0.002; Intact-lesion: *p* = 0.001; Genotype × Intact-lesion: *p* = 0.17, Figure 3F). Subsequent, *post hoc* analysis revealed that both intact and lesioned GPR37 KO striata displayed significant increased levels of GABA (Intact: *p* = 0.03; Lesion: *p* = 0.001, Figure 3F). The same analysis showed that the GABA amount was significantly increased by the lesion only in the GPR37 KO mice (WT: *p* = 0.11; KO: *p* = 0.004, Figure 3E).

### Striatal Slices From GPR37 KO Mice Exhibit Reduced LTP Development

**FIGURE 4:**
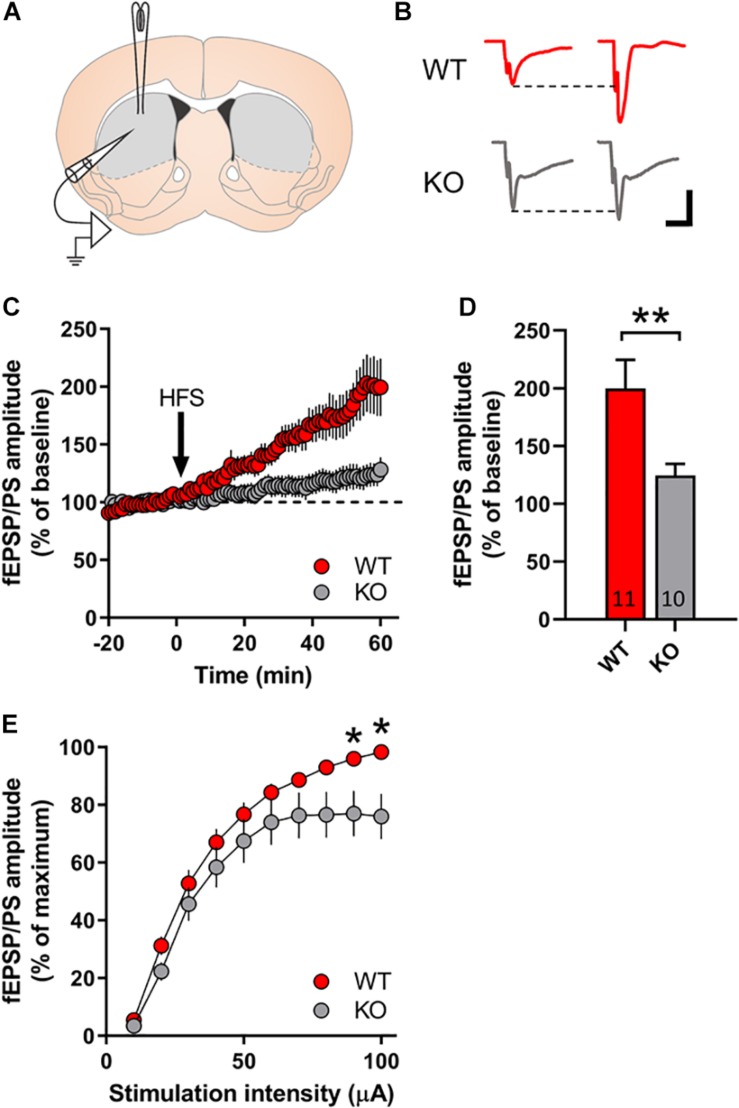
GPR37 KO striata display reduced LTP formation. **(A)** Schematic representation of the recording set up in coronal sections of dorsal striatum. **(B)** Representative traces in WT (red) and GPR37 KO (gray) of fEPSPs before and after high frequency stimulation. **(C)** Line graph showing the fEPSP/PS amplitude over time for WT (red) and GPR37 KO (gray). **(D)** Bar graph showing the average fEPSP/PS amplitude in the two genotypes. **(E)** Input/Ouput curves illustrating the averaged amplitude of the fEPSP/PS evoked by increasing stimulation intensities in WT and GPR37 KO animals. The *n* for each group is denoted in the corresponding bar. For **(E)** WT: *n* = 17; KO: *n* = 22. Data are expressed as mean ± SEM. *p < 0.05, **p < 0.01, Student’s *t*-test.

We investigated the possibility of GPR37 to regulate striatal LTP formation, which is a strongly DA dependent process (Schotanus and Chergui, 2008). For this reason, we used the coronal striatal sections from either WT or GPR37 KO mice (Figure 4). We observed a significant genotype effect upon LTP formation in striatum (*p* = 0.006, Figure 4D). Indeed, we observed a 60% LTP amplitude reduction in GPR37 KO striata (Figure 4D). Considering that corticostriatal LTP is highly dependent on DA terminal functionality, we assume that these findings further indicate a possible DA neuron dysfunction. We also examined whether the basic properties of glutamatergic synaptic transmission was altered. We found that there were significant different of input/output curves of fEPSP/PSs amplitude when the stimulation intensity at 90 μA and 100 μA between the WT and GPR37 KO mice (90 mA: *p* = 0.026; 100 mA: *p* = 0.01).

### GPR37 KO Mice Have Altered Levels of Glutamate Receptor Subunit Levels

**FIGURE 5:**
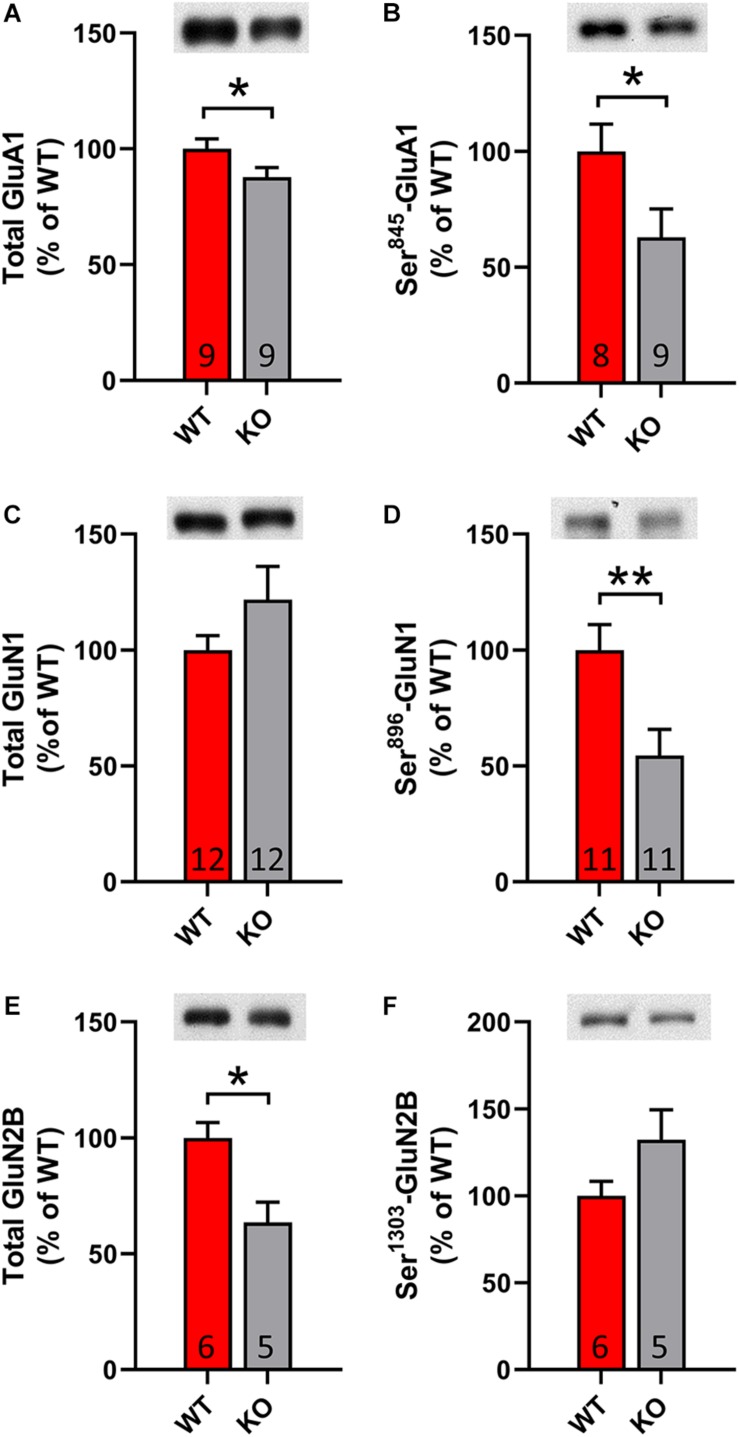
GPR37 KO show altered striatal glutamate receptor subunit content. **(A–F)** Bar graphs showing the total or phosphorylated protein level for AMPA receptor subunit **(A,B)** and NMDA receptor subunits **(C–F)** in WT and GPR37 KO striata. The *n* for each group is denoted in the corresponding bar. Data are expressed as mean ± SEM. *p < 0.05, **p < 0.01, Student’s *t*-test or Mann-Whitney test.

It is known that DA dependent LTP is accompanied by increased membrane trafficking of AMPA and NMDA receptors. In order to validate the identified changes upon electrophysiology, we conducted western blot analysis with lysates from striatal tissue. Remarkably, we observed a significant decrease in total and phosphorylated levels of AMPA receptor subunit 1 (GluA1) and NMDA receptor subunit 2B (GluN2B) (*p* = 0.03; *p* = 0.047; *p* = 0.02, Figures 5A,B,E,F). Furthermore, we identified significantly decreased phosphorylation of NMDA receptor subunit 1 (GluN1) at the site Ser^896^, which indicates less membrane trafficking of the receptor (*p* = 0.01, Figures 5C,D). In summary, our results denote that GPR37 striata undergo a synaptic plasticity defect.

## Discussion

In the present study we show that the absence of GPR37 in mice is linked to age-related motor deficits due to GABAergic and dopaminergic neuronal dysfunctions. It is known that GPR37 plays differential role cell viability as it has both neurotoxic and neuroprotective properties (Leinartaité and Svenningsson, 2017). It has been shown that GPR37 trafficking to the cell membrane promotes cell viability (Lundius et al., 2014). There is a strong expression of GPR37 on myelin-enriched areas (Yang et al., 2016; Smith et al., 2017; Jolly et al., 2018). In these studies, it is claimed that GPR37 is expressed in mature oligodendrocytes and potentiates resistance to demyelination. A protective role of GPR37 is further supported by stroke models where GPR37KO animals showed exacerbated inflammatory process after injury (Bang et al., 2018; Qu and Caterina, 2018; McCrary et al., 2019). In contrast, previous studies in GPR37 KO mice have described a resistant phenotype to the experimental PD toxin MPTP and no effect of aging in locomotion (Marazziti et al., 2004; Mandillo et al., 2013). This neuroprotective effect can be supported by the fact that GPR37 is able to form intracellular aggregated and cause ER stress (Kitao et al., 2007; Dusonchet et al., 2009; Marazziti et al., 2009). Here we used GPR37 KO animals backcrossed against a C57Bl6 background which is different than the aforementioned studies. Furthermore, we used another common experimental PD model, intrastriatal 6-OHDA. We found that GPR37 KO mice displayed lower levels of striatal DAT after the intrastriatal injection of 6-OHDA compared to the WT mice. It has been reported that GPR37 KO mice have reduced DAT and DA levels (Marazziti et al., 2004, 2007). However, we did not observe any alterations regarding DAT and DA in the intact side of the GPR37 KO mice. This inconsistency may reflect the methodological differences between autoradiography and WB but also between MALDI-MSI and HPLC (Marazziti et al., 2004, 2007; Imai et al., 2007). Furthermore, we describe a diffuse expression pattern of GPR37 in SNc compared to robust enrichment of the protein in the region by Imai et al. (2001). Since the aforementioned study used an immunohistochemical approach there is possibility that the GPR37 mRNA and protein may display partial overlap (Imai et al., 2001). Additionally, our ISH autoradiograph shows almost identical pattern with immunohistoblots of Morato et al. where they examined the specificity of the antibody with the same GPR37 KO line that we used (Morató et al., 2017). This fact strengthens the validity of our results where GPR37 is mainly enriched in myelinated areas while it shows a more diffused expression in SNc.

We studied striatal GABA levels and found that the intact striatum demonstrated an increase in GABA content which was potentiated upon 6-OHDA lesioning in GPR37 KO mice. One of the most well-known states that lead to considerable increase in striatal GABA is DA deafferentation as in PD (Kish et al., 1986). Both human and animal studies have repeatedly show increased GABA concentration in the striata of the affected individuals (Kish et al., 1986; Heo et al., 2017; Huang et al., 2019). Regarding the rodent PD models, 6-OHDA lesions in medial forebrain bundle (MFB) show profound upregulation of GABA (Shariatgorji et al., 2014, 2019). In our model, we failed to show any lesion related change in the WT, possibly due to the smaller extent of dopamine loss due intrastriatal 6-OHDA lesion rather than MFB lesioning. However, GPR37 KO mice show a significantly stronger GABA signal after the lesion. These results agree with the cylinder test performance of these animals. Based on the aforementioned studies, we propose that these GABA changes may stem from DAergic neuron malfunctioning and predispose to a worse behavioral outcome. GPR37 has been reported to interact with adenosine 2A receptor (A2AR) of D2 + MSNs and reduce its surface expression (Morató et al., 2017). This scenario could explain the GABA results as the lack of GPR37 would lead to aberrant A2AR surface expression and enhanced activity of D2 + MSNs (Svenningsson et al., 1999). Due to this fact it is difficult to determine if the primary cause of GABA upregulation is the enhanced A2AR signaling or the SNc neuronal dysfunction. Interestingly, A2AR antagonists are stimulating locomotion and several studies have shown that they have neuroprotective properties against DA neuron degeneration (Chen et al., 2001; Kalda et al., 2006; Xu et al., 2016). There is also evidence that GPR37 interacts with D2 and enhances its binding affinity to agonists (Dunham et al., 2009; Sokolina et al., 2017). Consistent with our present data, such potentation of D2 mediated signaling resulting in locomotion would be diminished in the absence of GPR37.

After striatal DAergic denervation, there is a simultaneous reduced and increased activity of D1 + MSNs and D2 + MSNs, respectively (Ryan et al., 2018). This is ascribed to the synchronized hampering of corticostriatal input to D1 + MSNs and the augmentation of cortical afferent drive to D2 + MSNs (Shepherd, 2013). LTP induction in MSNs after local high frequency stimulation within striatum is strongly dependent upon DA terminal integrity (Skiteva et al., 2018). The DA-dependency in this method of striatal LTP induction, derives from the fact that endostriatal high frequency stimulation induces DA release from the nigrostriatal terminals in a both direct and indirect manner (Schotanus and Chergui, 2008; Zhang et al., 2014). The indirect DA terminal activation is mediated through the stimulation of cholinergic interneurons (Sulzer et al., 2016). Consequently, the observed LTP in this electrophysiological approach may mainly depict the D1 + MSN corticostriatal synapses. We took advantage of this electrophysiological approach in order to evaluate whether GPR37 KO exhibit DA axon activity deficits. Interestingly, we detected a profound suppression of LTP amplitude in GPR37 KO striatal slices that agrees with the MALDI-MSI results on GABA levels. By using this method, we suggest a possible DA driven mechanism that explains the observations that we made in GPR37 KO mice. These data are in agreement with previous studies showing reduced locomotor response of GPR37 KO to the DAergic psychostimulant, amphetamine (Marazziti et al., 2004; Imai et al., 2007). Previous studies have studied LTP induction in GPR37 KO hippocampi but not striata (Cunha et al., 2008). There is evidence that GPR37 KO mice display normal LTD induction (Morató et al., 2019). However, LTD process is potentiated in GPR37 KO striata after the chronic administration of A2AR antagonist (Morató et al., 2019). Our WB analysis revealed a decrease in total GluA1 and GluN2B but also in phosphorylated GluA1 and GluN1. These results indicate less membrane availability of AMPARs and NMDARs in MSNs consistent with reduced corticostriatal LTP. Moreover, the surface availability of both these ionotropic glutamate receptors is critically related to D1 receptor activation (Surmeier et al., 2007). Specifically, the D1 driven cAMP accumulation leads to both phosphorylation and transcriptional changes that enhance receptors’ activity (Surmeier et al., 2007). There is evidence that GPR37 is expressed by striatal neurons and modulates their intracellular cAMP concentration, through its Gi-coupling. Indeed, viral transfection of opto-GPR37 in MSNs resulted in robust decrease in cAMP levels after light stimulation (Zheng et al., 2018). Considering these facts, we can conclude that our data on the phosphorylation states of GluA1 and GluN1 subunits cannot easily be explained by the intrinsic expression of GPR37 in striatum and requires further investigation.

## Conclusion

In conclusion, we describe here that elderly GPR37 KO mice have a reduced locomotor repertoire. This is preceeded by increased striatal GABA, altered NMDA and AMPA subunit composition and reduced corticostriatal synaptic plasticity. GPR37 KO mice have a heightened response to intrastriatal 6-OHDA lesioning with enhanced reduction of DAT and increased striatal GABA levels along with worse cylinder test score. Our data suggests that the absence of GPR37 may be a risk factor for dopaminergic neuron dysfunction and subsequent aging and parkinsonian related motor deficits.

## Data Availability Statement

All datasets generated for this study are included in the article/supplementary material.

## Ethics Statement

The animal study was reviewed and approved by the Local Ethical Committee at Karolinska Institute (N105/16).

## Author Contributions

XZ, IM, and EF performed the experiments, analyzed the data, and wrote the manuscript. PA, KC, and PS designed the study and wrote the manuscript.

## Conflict of Interest

The authors declare that the research was conducted in the absence of any commercial or financial relationships that could be construed as a potential conflict of interest.
